# Editorial: Community series in reducing adverse effects of cancer immunotherapy, volume II

**DOI:** 10.3389/fimmu.2025.1740320

**Published:** 2025-11-28

**Authors:** Lara Luisa Valerio de Mello Braga, Leonardo Vinícius Barbosa, Cleber Machado de Souza, Daniele Maria-Ferreira

**Affiliations:** 1 Faculdades Pequeno Príncipe, Curitiba, Brazil; 2 Instituto de Pesquisa Pelé Pequeno Príncipe, Curitiba, Brazil

**Keywords:** cancer immunotherapy, cancer treatment, cancer treatment toxicity, immunotherapy side effects, immunotherapy tolerability, immunotherapy toxicity

Chemotherapy and radiotherapy are effective but often cause adverse effects, requiring careful management to ensure treatment adherence and patient well-being. This Research Topic is the second volume of the “*Community series in reducing adverse effects of cancer immunotherapy*.”

Zhang et al. retrospectively reviewed six cases of a rare immune-related adverse effect: hepatic cavernous hemangioma induced by camrelizumab, a PD-1 inhibitor. All patients developed hepatic lesions consistent with cavernous hemangiomas, sometimes resulting in severe complications such as intra-abdominal bleeding. Lesion regression after discontinuation of camrelizumab in two cases supports a causal association. The persistence of reactive cutaneous capillary endothelial proliferation and the lack of effect from other agents, such as bevacizumab, suggest a unique pathogenic mechanism. This series underscores the need for careful monitoring of atypical hepatic lesions in patients treated with PD-1 inhibitors.

Tian et al. performed a bibliometric and visual analysis of chemotherapy-induced nausea and vomiting (CINV) research from 2004 to 2023, covering 734 publications from 61 countries. The University of Toronto, Merck & Co., Sun Yat-sen University, and Helsinn Healthcare SA were identified as leading contributors, while Supportive Care in Cancer was the most influential journal. Among 3,917 contributing authors, Rudolph M. Navari, Matti Aapro, Shimokawa Mototsugu, and Lee Schwartzberg were the most prolific. The analysis highlights ongoing research into CINV mechanisms and management strategies, outlining current frontiers and emerging trends in the field.

Katsin et al. reported a case of stage IV primary mediastinal B-cell lymphoma successfully treated with intrathecal methotrexate, cytarabine, and dexamethasone as first-line therapy for grade IV immune effector cell-associated neurotoxicity syndrome (ICANS) related to CD19 CAR-T cell treatment. Rapid resolution of ICANS to grade 0 allowed discontinuation of systemic corticosteroids, preserving CAR-T cell function. The patient achieved complete clinical and radiologic remission and remained disease-free after nine months, suggesting that intrathecal chemotherapy may be an effective first-line option for managing severe ICANS.

Gan et al. retrospectively analyzed medical records of 228 non-small cell lung cancer patients to evaluate the effects of sequential first- to third-generation EGFR-TKI therapy on corrected QT (QTc) intervals. They found that first-generation EGFR-TKIs prolonged QTc, with further prolongation observed after subsequent third-generation therapy. QT and QTc intervals increased paradoxically with rising heart rate, a trend more pronounced after sequential treatment. Pericardial effusion and low serum potassium levels were identified as independent predictors of additional QTc prolongation.

Lang et al. investigated the effects of cynaroside on methotrexate (MTX)-induced intestinal inflammation in Sprague–Dawley rats treated with 7 mg/kg MTX for 3 days, followed by cynaroside administration. MTX caused weight loss, reduced food intake, and increased disease activity, while cynaroside mitigated these effects. Treatment reduced inflammatory cell infiltration, restored goblet cell numbers, and decreased serum TNF-α, IL-1β, IL-18, and intestinal CD68-positive cells. Cynaroside also downregulated NLRP3, cleaved caspase-1, and cleaved IL-1β expression. The findings indicate that cynaroside alleviates MTX-induced intestinal injury by suppressing NLRP3 inflammasome activation.

Zhang et al. reported a case of secondary adrenocortical insufficiency after treatment with retifanlimab, a PD-1 inhibitor, in a 73-year-old woman with postoperative endometrial cancer. Six months after therapy, the patient developed anorexia, nausea, vomiting, malaise, electrolyte disturbances, and decreased serum adrenocorticotropin and cortisol levels. Symptoms resolved and electrolytes normalized after three days of intravenous hydrocortisone (200 mg per day). This case underscores the importance of promptly recognizing immune-related endocrine adverse events during immunotherapy and evaluating endocrine function in patients with nonspecific gastrointestinal symptoms.

Wassie et al. evaluated the prevalence and determinants of baseline anemia among adult cancer patients treated at oncology units of Northwest Amhara Comprehensive Specialized Hospitals, Ethiopia, in 2021. Baseline anemia was highly prevalent. Patients with low body mass index, and individuals diagnosed with stage III cancer were at greater risk. The authors recommend prioritizing early screening and management of anemia, particularly in these high-risk groups, to improve treatment tolerance and overall quality of life.

Zhao et al. retrospectively analyzed 208 esophageal squamous cell carcinoma (ESCC) patients treated with neoadjuvant immunochemotherapy to assess associations between the Systemic Inflammation Prognostic Score (SIPS), treatment-related adverse events (TRAEs), and outcomes. Patients classified as SIPS2 had lower BMI, more advanced disease, and lower pathological complete response rates. Serious TRAEs were more frequent in SIPS2 and were independently associated with higher SIPS (OR = 4.04, P = 0.010). Three-year disease-free survival (DFS) and overall survival (OS) declined with increasing SIPS. SIPS correlated with DFS but not OS, indicating its value as a predictor of serious TRAEs and prognosis in ESCC.

Di et al. reported a case of a 27-year-old woman with lung adenocarcinoma who developed urinary irritation after receiving pembrolizumab combined with chemotherapy. Repeated urine cultures were negative, and ultrasound revealed a 5.6 × 4.5 mm lesion on the right bladder wall. Transurethral resection (TURBT) showed infiltration of CD3^+^CD8^+^ lymphocytes, plasma cells, and eosinophils, with lymphoid follicle formation in the bladder mucosa. This case represents non-bacterial cystitis induced by immunotherapy.

Bai et al. studied 107 lung cancer patients receiving platinum-based chemotherapy to assess the relationship between physical activity and nausea or vomiting. More than half experienced these symptoms, while 50.5% had moderate to high activity levels. Higher activity was negatively correlated with symptom severity (P < 0.05). Multivariate analysis identified gender, alcohol history, and activity level as significant factors, with moderate to high physical activity serving as a protective factor against chemotherapy-induced nausea and vomiting.

Zhou et al. described a 69-year-old man with recurrent esophageal cancer who developed asymptomatic myocarditis after three cycles of sintilimab plus nab-paclitaxel. Elevated cardiac troponin I confirmed the diagnosis, and methylprednisolone improved markers but caused pneumonia and septic shock. A literature review identified 14 similar cases across various cancers. This is the first report of sintilimab-induced myocarditis in esophageal cancer, highlighting the need for cardiac monitoring and awareness of steroid-related adverse effects.

Pu et al. reported a case of pulmonary sarcomatoid carcinoma with high PD-L1 expression that progressed rapidly after bronchoscopic intervention and first-line therapy. The patient received tislelizumab plus anlotinib as second-line treatment, achieving near-partial remission after one cycle and continued tumor shrinkage until fatal hemoptysis occurred. This is the first reported case of fatal hemoptysis with this regimen, highlighting both the potential efficacy and serious risk of combining tislelizumab with anlotinib in centrally located PSC.

Yang et al. analyzed FAERS data to determine whether statin use increases immune-related adverse events (irAEs) in patients receiving immune checkpoint inhibitors (ICIs). Of 145,214 ICI-treated patients, 9,339 used statins. Statin use was associated with a higher risk of irAEs (OR = 1.199, 95% CI: 1.141–1.261; FDR p < 0.001), particularly in lung, pancreatic, and renal cancers. This association persisted across different ICIs. The study suggests that statin use may increase the risk of irAEs, highlighting the need for careful evaluation of statin therapy in cancer patients undergoing ICI treatment.

Chen et al. analyzed FAERS data from 2019 to 2024 to assess ocular adverse events associated with antibody–drug conjugates (ADCs). Among 2,686 reports, Tisotumab vedotin showed the strongest signals, followed by trastuzumab emtansine and enfortumab vedotin, while gemtuzumab ozogamicin showed minimal toxicity. Most adverse events involved corneal or neuromuscular disorders, with enfortumab vedotin showing the earliest onset at 12.5 days. The study reveals key associations and previously unreported ocular safety signals for ADCs.

Ji et al. reported a rare case of sintilimab-induced ureteritis and cystitis in a 55-year-old man with gastric cancer. The patient developed renal colic, hematuria, and hydronephrosis that did not respond to standard therapy. Bilateral ureteral stenting relieved symptoms and improved renal function, allowing successful surgery. This case suggests that ureteral stenting may be an effective corticosteroid-free option for ICI-induced ureteritis and cystitis.

Wang et al. reported a case of a lung cancer patient who developed grade 4 immune-related hypophysitis after chemotherapy combined with the PD-1 inhibitor sintilimab. The patient experienced fatigue, behavioral changes, somnolence, and diabetes insipidus. Symptoms improved with dexamethasone pulse therapy followed by tapering prednisone, and sintilimab was discontinued. The patient’s condition stabilized, and urine output returned to normal.

Chen et al. analyzed SEER data from 23,827 patients who underwent first primary lung cancer (FPLC) surgery, of whom 5,302 developed second primary lung cancer (SPLC). FPLC radiotherapy was associated with poorer overall and cancer-specific survival, while chemotherapy improved overall survival. Prior chemoradiotherapy reduced survival in SPLC, though subsequent SPLC radiotherapy improved outcomes, especially in patients without previous chemoradiotherapy. The study highlights that prior FPLC treatment influences SPLC prognosis and should guide individualized management.

Kassie et al. conducted a retrospective study of 354 cancer patients (mean age 41.3 ± 16.7 years) to compare blood cell profiles before and after chemotherapy. Anemia prevalence increased from 25.1% to 35.5%, neutropenia from 22.3% to 28%, and lymphopenia from 16.4% to 17.5%. Mean platelet, RBC, and WBC counts decreased by 23.5 × 10^3^/mm^3^, 0.63 × 10^6^/mm^3^, and 2.49 × 10^6^/mm^3^, respectively. Chemotherapy significantly reduced hematological parameters, indicating the need for replacement therapy after treatment.

Zhou et al. conducted a systematic review and meta-analysis of 28 high-quality studies to identify risk factors for checkpoint inhibitor pneumonitis (CIP) in lung cancer patients treated with ICIs. They identified 20 risk factors, including advanced age, male sex, smoking, preexisting lung disease, thoracic radiotherapy, squamous histology, PD-1 inhibitor use, and elevated PD-L1 expression. Sensitivity analyses confirmed the consistency of the results, and no publication bias was detected. The study highlights the importance of early screening and intervention in high-risk patients to reduce CIP incidence and improve outcomes.

Yang et al. reviewed the safety and efficacy of immune checkpoint inhibitors (ICIs) in lung cancer patients with preexisting autoimmune diseases (PAD), emphasizing current evidence, optimal treatment timing, and predictive biomarkers for immune-related adverse events (irAEs). They highlight the need for prospective studies to guide ICI use, identify at-risk patients, manage ICI resumption after irAEs, and develop therapies that control both cancer and PAD.

Liu et al. analyzed FAERS data (2004–2024) to characterize the profiles and risk factors of Stevens-Johnson syndrome (SJS) and toxic epidermal necrolysis (TEN) associated with anticancer therapies. Among 3,471 unique cases involving 159 drug pairs, 31 drugs showed significant associations. Targeted therapies accounted for 35.9%, chemotherapies for 35.5%, and immunotherapies for 21.5% of cases, with a median onset of 17 days. Age over 65, female sex, and 10 specific anticancer drugs were significant risk factors. The study provides real-world evidence on the burden of SJS/TEN, highlighting the need for improved prevention and management strategies.

This Research Topic has produced novel and impactful findings that significantly advance current understanding in this research field. We sincerely thank all authors, reviewers, and editors for their essential contributions to the progress and dissemination of this scientific work.

